# Reasons for COVID-19 Non-Vaccination from 2021 to 2023 for Adults, Adolescents, and Children

**DOI:** 10.3390/vaccines12060568

**Published:** 2024-05-22

**Authors:** Kimberly H. Nguyen, Yingjun Bao, Julie Mortazavi, Laura Corlin, Jennifer D. Allen

**Affiliations:** 1 Hubert Department of Global Health, Emory University Rollins School of Public Health, Atlanta, GA 30322, USA; 2 Department of Public Health & Community Medicine, Tufts University School of Medicine, Boston, MA 02111, USA; 3 Department of Civil and Environmental Engineering, Tufts University School of Engineering, Medford, MA 02155, USA; 4 Department of Community Health, Tufts School of Arts and Sciences, Medford, MA 02115, USA

**Keywords:** COVID-19 vaccination, vaccine hesitancy, mistrust, vaccine confidence, non-vaccination, attitudes, behaviors, beliefs, children, adults

## Abstract

Understanding how attitudes and beliefs about COVID-19 vaccination have changed over time is essential for identifying areas where targeted messaging and interventions can improve vaccination confidence and uptake. Using data from multiple waves of the nationally representative U.S. Census Bureau’s Household Pulse Survey collected from January 2021 to May 2023, we assessed reasons for the non-vaccination of adults, adolescents, and children using the Health Belief Model as the framework for understanding behavior. Among unvaccinated adults, perceived vulnerability increased from 11.9% to 44.1%, attitudinal factors/mistrust increased from 28.6% to 53.4%, and lack of cue to action increased from 7.5% to 9.7% from January 2021 to May 2022. On the other hand, safety/efficacy concerns decreased from 74.0% to 60.9%, and logistical barriers to vaccination decreased from 9.1% to 3.4% during the same time period. Regarding reasons for non-vaccination of youth, perceived vulnerability increased from 32.8% to 40.0%, safety/efficacy concerns decreased from 73.9% to 60.4%, and lack of cue to action increased from 10.4% to 13.4% between September 2021 and May 2023. While safety/efficacy concerns and logistic barriers have decreased, increases in perceived vulnerability to COVID-19, mistrust, and lack of cues to action suggest that more efforts are needed to address these barriers to vaccination.

## 1. Introduction

Despite the recommendations for and availability of COVID-19 vaccines, many adults and children are not up-to-date with all doses and boosters. For example, the latest data from 26 November–30 December 2023 indicate that 81.5% of adults ≥18 years in the U.S. were vaccinated (≥1 dose) and only 17.4% are up-to-date with COVID-19 vaccines (i.e., received the updated bivalent booster vaccine) [1]. Among children aged 6 months to 17 years, over one-half (52.2%) were vaccinated (≥1 dose), and only 7.5% are up-to-date with COVID-19 vaccines [2]. During the same period, there has been an increase in COVID-19 emergency department visits and hospitalizations, prompting concern about the increase in COVID-19 infections and low rates of vaccination [3]. A nationally representative study of 5458 adults from 1 May to 30 June 2021 found that 76.1% of cases of non-vaccination of COVID-19 vaccines was attributed to vaccine hesitancy, suggesting that hesitancy, rather than access or other factors, was the largest barrier to receiving the recommended COVID-19 vaccines at that time [4]. 

Numerous studies have examined factors associated with adult and parental intentions to pursue COVID-19 vaccinations [5,6,7,8,9,10,11,12,13]. However, the vast majority of studies have examined vaccination coverage or intent at one point in time during the pandemic [14,15,16,17]. Given that COVID-19 vaccine attitudes and behaviors likely change over time, there is a need for studies that examine vaccine attitudes and behaviors. Previous studies have consistently shown that concerns about vaccine safety and efficacy, as well as mistrust in the government and COVID-19 vaccines, are correlated with vaccine behaviors and intentions [18,19,20,21,22,23].

The goal of this study was to utilize concepts from the Health Belief Model (HBM) to examine factors associated with and changes in reasons for non-vaccination of adults and children among a large, nationally representative survey of U.S. households [24]. The HBM posits that behavioral action is most likely when perceived vulnerability is high, perceived benefits are great, perceived barriers are low, and there are cues to action that prompt the behavior. Although the HBM also includes self-efficacy with regard to performing the behavior, this was not deemed directly relevant to the current inquiry, since individuals do not vaccinate themselves or their children. Examining factors associated with reasons for non-vaccination using a theoretical framework is important for targeting mechanisms of behavioral change, and a recent systematic review found that HBM constructs significantly predicted COVID-19 vaccine hesitancy [18,25]. Moreover, examining changes in reasons for non-vaccination throughout the pandemic can help identify evolving behaviors, attitudes, and perceptions to make appropriate predictions for developing messages and interventions to improve vaccination uptake and confidence. 

## 2. Methods

### 2.1. Study Design

Since 2020, the U.S. Census Bureau has conducted the Household Pulse Survey (HPS), which is a nationally representative cross-sectional household survey of adults aged ≥18 years, to assess the impact of the COVD-19 pandemic on U.S. households. In 2021, COVID-19 questions such as coverage, intent, and reasons for non-vaccination were added to the survey. The survey design of the HPS has been described previously [26]. Non-institutionalized adults aged ≥18 years in the United States were selected from the Census Bureau’s Master Address File and contacted via email and/or text to complete the survey online using Qualtrics as a data collection platform. Datasets from the following survey waves were used in this study: 6 January 2021 to 18 January 2021 (response rate = 6.4%), 28 April 2021 to 10 May 2021 (response rate = 7.4%), 1 September 2021 to 13 September 2021 (response rate = 6.0%), 29 December 2021 to 10 January 2022 (response rate = 7.2%), 27 April 2022 to 9 May 2022 (response rate = 5.8%), 14 September 2022 to 26 September 2022 (response rate = 4.7%), 9 December 2022 to 19 December 2022 (response rate = 6.7%), and 26 April 2023 to 8 May 2023 (response rate = 5.5%). Per the Emory University Institutional Review Board’s determination assessments, this study is not considered human subject research.

### 2.2. Reasons for Non-Vaccination

Among those who had not been vaccinated or received all required doses, respondents were asked reasons for not getting vaccinated or receiving all required doses of the vaccine. Based on the HBM, the responses were combined into five categories: (1) perceived vulnerability to COVID-19, (2) safety/efficacy concerns, (3) attitudinal factors/mistrust, (4) logistical barriers, and (5) lack of cues to action. ‘Perceived vulnerability’ was determined by agreement with the following responses as reasons for non-vaccination: (1) I do not think COVID-19 is that big of a threat, (2) I do not believe I need a COVID-19 vaccine, and (3) I believe one [vaccine] dose is enough to protect me (only asked among those who did not receive all required doses). Safety/efficacy concerns were determined by agreement with the following responses: (1) I am concerned about possible side effects of a COVID-19 vaccine, (2) I do not know if a COVID-19 vaccine will protect me, (3) I plan to wait and see if it is safe and may receive it later, and (4) I experienced side effects from the dose of COVID-19 vaccine I received. Attitudinal factors/mistrust were determined by agreement with the following responses: (1) I do not trust COVID-19 vaccines, and (2) I do not trust the government. Logistical barriers were determined by agreement with the following responses: (1) It is hard for me to get a COVID-19 vaccine, and (2) I am concerned about the cost of a COVID-19 vaccine. Finally, lack of cue to action was determined by agreement with the following response: my doctor has not recommended it. 

Among adults who received ≥1 COVID-19 doses but did not receive the updated bivalent booster vaccine, perceived vulnerability was determined by agreement with the following responses: (1) I think I have enough immunity to COVID-19 from prior doses of the vaccine, (2) I am not worried about having COVID-19, and (3) I already had COVID-19. Safety/efficacy concerns were determined by agreement with the following response: I experienced side effects from my previous dose(s) of the COVID-19 vaccine. Finally, lack of cue to action was determined by agreement with the following responses: (1) my doctor has not recommended it, and (2) I am not required to get a COVID-19 booster (by my work or school).

Among adults who lived with children in the household (hereafter referred to as “parents” for simplicity, even though they may include grandparents, guardians, or anyone else who lives in the household), they were asked whether children in the household received COVID-19 vaccination. Among parents whose children did not receive COVID-19 vaccination, they were asked reasons for not vaccinating their children. These reasons were categorized as follows: perceived vulnerability, which was determined by agreement with the following responses: (1) I do not believe children need a COVID-19 vaccine, and (2) the children in this household are not members of a high-risk group. Safety/efficacy concerns were determined by agreement with the following responses: (1) [I am] concern[ed] about possible side effects of a COVID-19 vaccine for children, (2) [I am] not sure if a COVID-19 vaccine will work for children, and (3) [I] plan to wait and see if it is safe and may receive it later. Attitudinal factors/mistrust were determined by agreement with the following responses: (1) [I] do not trust COVID-19 vaccines, and (2) [I] do not trust the government. Logistical barriers were determined by agreement with the following responses: (1) concern about missing work to have the children vaccinated, (2) unable to get a COVID-19 vaccine for children in this household, and (3) concern about the cost of a COVID-19 vaccine. Finally, cues to action were determined by agreement with the following response: the children’s doctor has not recommended it. 

### 2.3. Statistical Analysis

Sociodemographic characteristics were assessed for all adults and those living with children in the household. We considered the respondents’ age group [18–29, 30–39, 40–49, 50–64, ≥65 years], gender [men, women], race/ethnicity [Hispanic, non-Hispanic (NH) Asian, NH Black, NH White, NH another/multiracial], highest educational attainment [high school equivalent or less, some college or Associate’s degree, Bachelor’s degree, graduate degree], annual household income [<USD 35,000, USD 35,000–49,999, USD 50,000–74,999, ≥USD 75,000, did not report], health insurance status [covered, not covered], respondent history of COVID-19 infection [yes, no], and COVID-19 vaccination status [yes, no]. 

Changes in reasons for non-vaccination were assessed between 6 January 2021 and 9 May 2022 for vaccines for adults and 1 September 2021 to 8 May 2023 for vaccines for children and adolescents, and the prevalence of reasons for under-vaccination was assessed for adults using data from 26 April 2023 to 8 May 2023. Using the latest survey waves, factors associated with reasons for non-vaccination based on the HBM (e.g., perceived vulnerability, safety/efficacy concerns, attitudinal factors/mistrust, and physician recommendation) for adults and children were assessed via adjusted odds ratios from multivariable regression models. Logistical barriers were not assessed in multivariable regression models due to the small sample size. Based on a review of the literature, independent variables in the model included respondent age group, gender, race/ethnicity, educational attainment, annual household income, insurance status, and prior COVID-19 infection. All results presented in the text are statistically significant at *p* < 0.05. Analyses accounted for the survey design and weights to ensure a representative sample in Stata (version 18.0).

## 3. Results

The sociodemographic characteristics of the sample are found in Table 1. Overall, for participants responding between 27 April 2022 and 9 May 2022, 62.0% of adults were non-Hispanic (NH) White, 39.0% had a high school education or less, 31.3% had an annual household income of ≥USD 75,000, and 84.4% had COVID-19 vaccination. The respondents from 26 April 2023 to 8 May 2023 living with children in the household were similar: 53.1% were non-Hispanic (NH) White, 41.0.0% had a high school education or less, 35.6% had an annual household income of ≥USD 75,000, and 77.7% had COVID-19 vaccination. 

**Figure 1:**
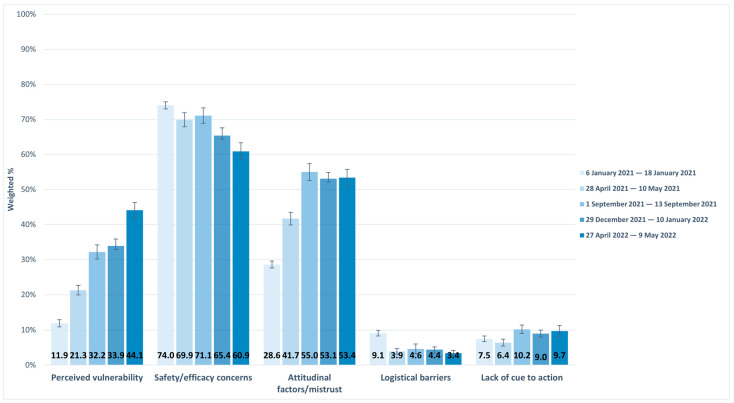
Distribution of change in reasons for COVID-19 non-vaccination among adults, Household Pulse Survey. 6 January 2021 to 9 May 2022.

Between 6 January 2021 and 9 May 2022 (Table 2, Figure 1), perceived vulnerability to COVID-19 increased by 32.1 percentage points (pp), from 11.9% in January 2021 to 44.1% in May 2022. Attitudinal factors/mistrust almost doubled from 28.6% to 53.4%, and cues to action increased from 7.5% to 9.7% during the same period. On the other hand, safety/efficacy concerns decreased from 74.0% to 60.9%, and logistic barriers decreased from 9.1% to 3.4%. 

Among those who were not vaccinated during the data collection period from 27 April 2022 to 9 May 2022, attitudes and beliefs toward non-vaccination were associated with several sociodemographic factors (Table 3). Perceived vulnerability was associated with being in a younger age group (e.g., 18–29 years versus ≥65 years: adjusted odds ratio [aOR] = 3.97, 95% confidence interval [CI]: 2.43, 6.50), identifying as a man (aOR = 1.97, 95%CI: 1.62, 2.41), having an education higher than a college degree (aOR = 2.65, 95%CI: 1.77, 3.95), and having an annual household income ≥USD 75,000 (aOR = 2.36, 95%CI: 1.76, 3.17). Those who had a prior COVID-19 infection were less likely to have perceived vulnerability (aOR = 0.70, 95%CI: 0.56, 0.87). Furthermore, safety/efficacy concerns were associated with younger age groups (18–29 age group: aOR = 2.79, 95%CI: 1.81, 4.31; and 30–39 years: aOR = 1.89, 95%CI: 1.32, 2.70). Those who identified as NH Black (aOR = 0.45, 95%CI: 0.29, 0.72) or Hispanic (aOR = 0.42, 95%CI: 0.29, 0.60) were less likely than those who identified as NH White to be associated with attitudinal factors/mistrust. Lastly, lack of cue to action was associated with adults who identify as NH White compared to NH Black (aOR = 2.31, 95%CI: 1.14, 4.67) and those who have an education higher than a college degree (aOR = 2.09, 95%CI: 1.21, 3.61). 

During April/May 2023, more than one-half (54.9%) of adults who received ≥1 dose of COVID-19 vaccine said that perceived vulnerability was a reason for not receiving the updated bivalent booster vaccine (Table 4). Approximately one-tenth (9.5%) of respondents stated that there were perceived barriers (e.g., side effects) to receiving the updated bivalent vaccine, and 19% had not received cues to action. 

**Figure 2:**
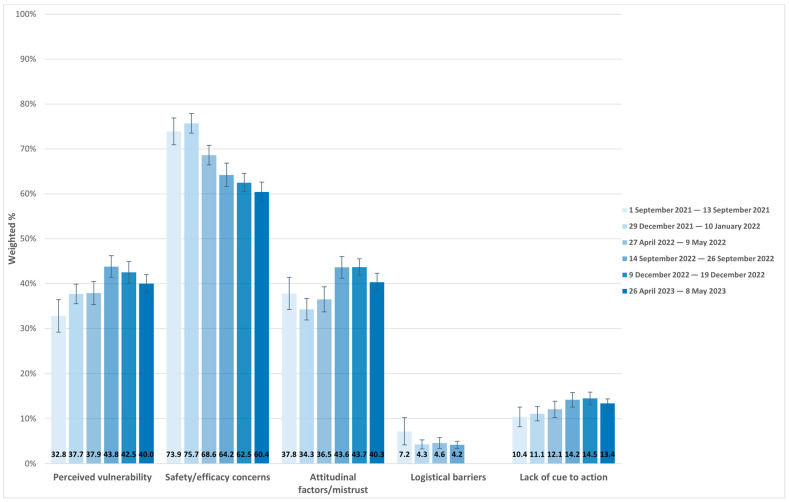
Distribution of change in reasons for COVID-19 non-vaccination of children, Household Pulse Survey 1 September 2021 to 8 May 2023.

Among parents of children, the perceived vulnerability of their child to COVID-19 increased from 32.8% in September 2021 (representing youth 12–17 only) to 40.0% in May 2023 (representing all age children; Table 5, Figure 2). In addition, safety/efficacy concerns decreased from 73.9% to 60.4%, and cue to action increased from 10.4% to 13.4% during the same period and in the same age groups. 

Perceived vulnerability of children to COVID-19 was associated with younger respondent age (e.g., 18–29 years: aOR = 4.05, 95%CI: 2.47, 6.63), identifying as a man (aOR = 1.26, 95%CI: 1.06, 1.48), having a college (aOR = 1.65, 95%CI: 1.26, 2.14) or higher than college degree (aOR = 1.62, 95%CI: 1.15, 2.28), and having an annual household income of ≥USD 75,000 (aOR = 1.94, 95%CI: 1.43, 2.64) (Table 6). Safety/efficacy concerns were associated with those who had a college (aOR = 1.54, 95%CI: 1.19, 2.00) or higher than college degree (aOR = 1.52, 95%CI: 1.08, 2.13), and those who had a prior COVID-19 infection (aOR = 1.38, 95%CI: 1.13, 1.67). Attitudinal factors/mistrust were associated with the respondent identifying as a man (aOR = 1.48, 95%CI: 1.20, 1.83). Lastly, lack of cue to action was associated with younger respondents (e.g., 18–29 years (aOR = 2.66, 95%CI: 1.23, 5.74), identifying as NH Black (versus NH Asian; aOR = 3.10, 95%CI: 1.07, 8.97), having a college (aOR = 1.50, 95%CI: 1.03, 2.16) or higher degree (aOR = 1.48, 95%CI: 1.05, 2.10), and having health insurance (aOR = 1.73, 95%CI: 1.12, 2.68).

## 4. Discussion

Attitudes and beliefs toward COVID-19 vaccination changed throughout 2021, 2022, and 2023. For example, perceived vulnerability (i.e., the belief that the vaccine is not needed or that COVID-19 is not that big of a threat) increased as a reason for non-vaccination of adults and youth. While safety/efficacy concerns of vaccines decreased, concerns about vaccine safety and efficacy continued to be the leading reason for non-vaccination, and mistrust of vaccines and of the government increased among unvaccinated adults. Furthermore, lack of cue to action (i.e., physician recommendation) for adult and childhood COVID-19 vaccinations increased during the pandemic, suggesting areas where targeted efforts can increase vaccination uptake and confidence. 

The interpretation of these findings was carried out in light of the HBM, which states that increased perceived vulnerability, decreased perceptions of barriers (e.g., safety and efficacy concerns, logistical barriers), and increased cues to action (physician recommendation) should translate into increased prevalence of vaccination and greater intentions to be vaccinated or to have one’s child vaccinated [27,28]. While we did not look at associations between HBM constructs and non-vaccination behaviors, the respondents attributed non-vaccination to these factors. Given strong secular trends and changes in eligibility for vaccination and boosters during the course of the pandemic, it would be problematic to state that these factors accounted for all of the changes in vaccination behaviors [29,30,31]. Nonetheless, a recent meta-analysis of 32 studies found that the variance explained by the HBM constructs on COVID-19 vaccination behaviors was “good”, with half of the studies finding that these constructs accounted for ≥50% [32]. Therefore, changes in HBM constructs, which have been found in numerous studies to be associated with COVID-19 vaccination and intentions, can provide some guidance to ongoing efforts to promote vaccination [29,30,31].

On the one hand, our findings suggest that efforts to educate the public that everyone is vulnerable to COVID-19 and that vaccination is widely available are working. However, ongoing concerns about vaccine safety and efficacy, coupled with increased mistrust among unvaccinated respondents, suggest that efforts to address these drivers of non-vaccination have not been very effective. Moreover, it is disappointing that provider recommendation, the most salient driver of vaccination [28], has not increased more substantially. Our study cannot point to reasons for these phenomena, but suggests there is still a dire need to identify effective interventions to address these issues. Lastly, targeted messages addressing barriers to non- or under-vaccination among specific populations are needed to protect against severe COVID-19 outcomes. 

Certain groups had higher perceived vulnerability, safety/efficacy concerns, and attitudinal factors/mistrust. For example, younger individuals, NH White respondents, and those with higher educational attainment were more likely to believe that COVID-19 is not a major concern and were less likely to receive a physician recommendation compared to their respective counterparts. While some of these results may be different from those conducted early in the pandemic, this demonstrates the changing patterns of hesitancy which evolved during the pandemic. These results are consistent with more recent studies that have shown hesitancy later on in the pandemic, and adds to the literature by showing reasons for hesitancy and its trends over time [33,34]. These results suggest that targeted approaches to address barriers to non-vaccination can improve uptake and confidence. 

### Limitations

This study adds new information to the large body of literature on factors’ association with COVID-19 vaccination, but there are limitations worth noting. While the Household Pulse Survey has a robust sample size and provides nationally representative data, the data may be subject to several limitations. First, although the sampling methods and data weighting were designed to produce nationally representative results, respondents might not be fully representative of the general U.S. adult population [26]. Second, the HPS had a low response rate (<10%), although the non-response bias assessment conducted by the Census Bureau found that the survey weights mitigated most of this bias [35]. Third, the cross-sectional nature of the survey prevents causal inferences. Fourth, vaccination status of respondents and their children/adolescents, and report of physician recommendation, were self-reported and may have been subject to recall or social desirability bias. Fifth, changes in attitudes/beliefs may be a result of changes in the sample. For example, as more people get vaccinated against COVID-19, the remaining unvaccinated individuals may be more hesitant, which may magnify some of the changes in perceptions and beliefs seen in this study. Sixth, this study did not contain all of the information necessary to assess Health Belief Model constructs (e.g., self-efficacy). Finally, this survey did not contain information on the child’s age, or number of children in the household, which may affect their reasons for non-vaccination.

## 5. Conclusions

Understanding changes in beliefs toward COVID-19 vaccination is important for developing effective and appropriate messaging and strategies to improve uptake among adults, adolescents, and children. Addressing the main barriers to non-vaccination and under-vaccination, which are safety/efficacy and perceived vulnerability, respectively, is needed to protect all populations from the harmful effects of COVID-19. Using proven strategies, such as working with trusted messengers to address misinformation and convey the importance of vaccines, can help address gaps and disparities in specific communities [36]. Furthermore, cues to action, such as provider recommendations, can promote confidence in vaccines [37]. As attitudes and behaviors toward COVID-19 vaccination continue to evolve, understanding the theoretical framework for these changes can help direct specific messaging and strategies to advance vaccination and confidence. Being up-to-date with all eligible vaccines is important for protection against severe disease for all populations.

## Figures and Tables

**Table 1:** Distribution of sociodemographic and other characteristics among adults and parents with children, Household Pulse Survey.

	Adults	Parents with Children
	27 April 2022 to 9 May 2022	26 April 2023 to 8 May 2023
	Weighted Percent (95% CI)	Weighted Percent (95% CI)
Overall (n)	61,767	20,203
Age Groups (in years)		
18–29	15.2 (14.7, 15.7)	14.9 (13.9, 15.9)
30–39	19.1 (18.5, 19.6)	28.8 (27.8, 29.8)
40–49	17.4 (16.9, 17.8)	29.1 (28.1, 30.0)
50–64	25.8 (25.5, 26.2)	18.5 (17.8, 19.2)
≥65	22.5 (22.3, 22.8)	8.7 (7.9, 9.5)
Gender		
Men	48.5 (48.2, 48.7)	45.3 (44.4, 46.2)
Women	51.5 (51.3, 51.8)	54.7 (53.8, 55.6)
Race/ethnicity		
Non-Hispanic White	62.0 (61.8, 62.2)	53.1 (52.2, 54.0)
Non-Hispanic Black	11.4 (11.3, 11.6)	13.2 (12.6, 13.8)
Hispanic	17.5 (17.2, 17.7)	22.7 (21.6, 23.8)
Non-Hispanic Asian	5.3 (5.1, 5.5)	5.9 (5.5, 6.4)
Non-Hispanic other/multiple races	3.8 (3.6, 4.1)	5.0 (4.5, 5.5)
Highest Educational Attainment		
High school or less	39.0 (39.0, 39.0)	41.0 (40.1, 42.0)
Some college or Associate’s	30.1 (30.1, 30.2)	29.3 (28.6, 30.0)
College graduate	16.9 (16.7, 17.2)	15.9 (15.4, 16.5)
Above college graduate	13.9 (13.7, 14.2)	13.7 (13.2, 14.3)
Annual Household Income		
<USD 35,000	19.4 (18.8, 20.0)	15.4 (14.3, 16.4)
USD 35,000–USD 49,999	9.0 (8.5, 9.4)	8.5 (7.7, 9.3)
USD 50,000–USD 74,999	12.8 (12.2, 13.3)	11.5 (10.7, 12.2)
≥USD 75,000	31.3 (30.9, 31.7)	35.6 (34.5, 36.7)
Did not report	27.5 (26.8, 28.2)	29.1 (27.7, 30.5)
Insurance status		
Insured	91.8 (91.1, 92.4)	90.8 (89.7, 92.0)
Not insured	8.2 (7.6, 8.9)	9.2 (8.0, 10.3)
Prior COVID-19 infection		
Yes	29.6 (28.8, 30.5)	59.9 (58.7, 61.1)
No	70.4 (69.5, 71.2)	40.1 (38.9, 41.3)
COVID-19 vaccination		
Yes	84.4 (83.7, 85.1)	77.7 (76.7, 78.8)
No	15.6 (14.9, 16.3)	22.3 (21.2, 23.3)
Children < 18 years living in the household		
Yes	37.5 (36.8, 38.3)	100%
No	62.5 (61.7, 63.2)	0%

Abbreviations: CI = confidence intervals.

**Table 2:** Changes in reasons for COVID-19 non-vaccination among adults, Household Pulse Survey.

	6 January 2021 to 18 January 2021 Phase 3.0	28 April 2021 to 10 May 2021 Phase 3.1	1 September 2021 to 13 September 2021 Phase 3.2	29 December 2021 to 10 January 2022 Phase 3.3	27 April 2022 to 9 May 2022 Phase 3.4	Prevalence Difference ^a^
	% (95% CI)	% (95% CI)	% (95% CI)	% (95% CI)	% (95% CI)	% (95% CI)
Unweighted N	24,111	11,475	6523	6915	6208	
Perceived vulnerability I do not think COVID-19 is that big of a threat ^b^ I do not believe I need a COVID-19 vaccine I believe one dose is enough to protect me ^b^	11.9 (11.0, 12.9)	21.3 (20.0, 22.7)	32.2 (30.2, 34.3)	33.9 (31.8, 36.0)	44.1 (41.9, 46.3)	32.1 (29.8, 34.5)
Safety/efficacy concerns Concerned about possible side effects of a COVID-19 vaccine I do not know if a COVID-19 vaccine will protect me (Phase 3.2, 3.3, 3.4) or I don’t know if a COVID-19 vaccine will work (Phase 3.0, 3.1) I plan to wait and see if it is safe and may get it later I experienced side effects from the dose of COVID-19 vaccine I received ^b^	74.0 (73.0, 75.0)	69.9 (68.0, 71.9)	71.1 (68.9, 73.3)	65.4 (63.1, 67.7)	60.9 (58.5, 63.4)	−13.1 (−15.8, −10.4)
Attitudinal factors/mistrust I do not trust COVID-19 vaccines I do not trust the government	28.6 (27.6, 29.5)	41.7 (39.9, 43.4)	55.0 (52.6, 57.3)	53.1 (51.3, 54.8)	53.4 (51.1, 55.8)	24.9 (22.1, 27.7)
Logistical barriers It is hard for me to get the vaccine^ b^ I am concerned about the cost of a COVID-19 vaccine	9.1 (8.4, 9.9)	3.9 (3.2, 4.6)	4.6 (3.2, 5.9)	4.4 (3.5, 5.2)	3.4 (2.5, 4.2)	−5.8 (−6.8, −4.7)
Lack of cue to action My doctor has not recommended it	7.5 (6.7, 8.4)	6.4 (5.4, 7.4)	10.2 (8.9, 11.4)	9.0 (7.9, 10.1)	9.7 (8.1, 11.3)	2.2 (0.2, 4.1)

Note: All values are weighted unless otherwise noted. Abbreviations: CI = confidence intervals. ^a^ difference in prevalence from Phase 3.0 to 3.4. ^b^ This response is only available in Phase 3.2, 3.3, 3.4.

**Table 3:** Bivariate and multivariable analyses of reasons for COVID-19 non-vaccination among adults, Household Pulse Survey, 27 April 2022 to 9 May 2022.

	Bivariate Analysis				Multivariable Analysis			
	Perceived Vulnerability	Safety/Efficacy Concerns	Attitudinal Factors/Mistrust	Lack of Cue to Action	Perceived Vulnerability	Safety/Efficacy Concerns	Attitudinal Factors/Mistrust	Lack of Cue to Action
	% (95%CI)	% (95%CI)	% (95%CI)	% (95%CI)	aOR (95%CI)	aOR (95%CI)	aOR (95%CI)	aOR (95%CI)
Overall	44.1 (41.9, 46.3)	60.9 (58.5, 63.4)	53.4 (51.1, 55.8)	9.7 (8.1, 11.3)				
Age Groups (in years)								
18–29	57.4 (50.9, 63.9) *	71.1 (64.4, 77.8) *	53.6 (46.6, 60.6)	11.9 (5.4, 18.5)	3.97 (2.43, 6.50)	2.79 (1.81, 4.31)	0.88 (0.57, 1.35)	2.14 (0.96, 4.80)
30–39	42.0 (38.0, 46.0)	60.8 (56.7, 64.9) *	54.4 (49.7, 59.2)	9.7 (7.4, 11.9)	1.56 (1.03, 2.37)	1.89 (1.32, 2.70)	1.17 (0.80, 1.70)	1.77 (0.92, 3.40)
40–49	40.1 (34.4, 45.9)	56.5 (51.2, 61.8)	52.1 (47.3, 56.9)	7.7 (5.4, 10.0)	1.55 (0.98, 2.45)	1.27 (0.88, 1.84)	0.91 (0.64, 1.29)	1.32 (0.69, 2.55)
50–64	41.1 (36.3, 45.8)	60.1 (55.9, 64.2) *	52.3 (48.0, 56.6)	9.7 (7.4, 12.0)	1.31 (0.86, 1.98)	1.49 (1.04, 2.14)	0.85 (0.57, 1.27)	1.41 (0.78, 2.56)
≥65 (ref)	36.4 (30.3, 42.4)	50.7 (45.6, 55.7)	55.9 (49.3, 62.5)	9.7 (5.2, 14.2)	1.0	1.0	1.0	1.0
Gender								
Men	52.1 (48.5, 55.7) *	60.6 (56.4, 64.8)	56.7 (52.4, 60.9) *	11.9 (8.7, 15.0) *	1.97 (1.62, 2.41)	0.97 (0.76, 1.26)	1.16 (0.88, 1.53)	1.50 (1.06, 2.13)
Women (ref)	36.1 (33.5, 38.7)	61.5 (58.9, 64.1)	49.6 (46.6, 52.7)	7.3 (6.3, 8.3)	1.0	1.0	1.0	1.0
Race/ethnicity								
Non-Hispanic White (ref)	50.3 (48.2, 52.4)	61.2 (58.4, 64.1)	59.9 (57.3, 62.5)	10.7 (9.1, 12.3)	1.0	1.0	1.0	2.31 (1.14, 4.67)
Non-Hispanic Black	27.0 (20.6, 33.4) *	58.1 (51.0, 65.3)	39.3 (32.2, 46.5) *	5.1 (2.6, 7.5) *	0.45 (0.28, 0.72)	0.88 (0.59, 1.31)	0.45 (0.29, 0.72)	1.0
Hispanic	33.4 (26.5, 40.3) *	61.9 (53.9, 69.9)	41.0 (33.9, 48.2) *	10.2 (4.2, 16.2)	0.39 (0.24, 0.63)	1.21 (0.78, 1.87)	0.42 (0.29, 0.60)	2.07 (0.75, 5.74)
Non-Hispanic Asian	45.2 (19.3, 71.1)	71.5 (52.9, 90.1)	38.8 (15.4, 62.1)	4.1 (0.0, 10.6)	0.72 (0.26, 2.01)	1.35 (0.56, 3.25)	0.39 (0.13, 1.13)	1.03 (0.11, 9.41)
Non-Hispanic other/multiple races	44.7 (36.4, 53.1)	58.6 (50.1, 67.0)	53.6 (44.6, 62.7)	8.7 (3.9, 13.5)	0.96 (0.59, 1.59)	1.67 (1.01, 2.77)	0.97 (0.60, 1.58)	2.46 (0.85, 7.11)
Highest Educational Attainment								
High school or less (ref)	39.0 (35.2, 42.9)	57.2 (52.7, 61.8)	51.9 (48.1, 55.7)	8.6 (6.2, 11.1)	1.0	1.0	1.0	1.0
Some college or Associate’s	47.9 (45.1, 50.7) *	65.4 (63.1, 67.7) *	55.1 (52.0, 58.2)	10.2 (8.4, 12.1)	1.30 (1.01, 1.69)	1.25 (0.98, 1.59)	1.07 (0.83, 1.40)	1.41 (0.97, 2.03)
College graduate	54.7 (49.8, 59.6) *	66.0 (61.1, 70.8) *	54.5 (50.4, 58.5)	12.0 (8.5, 15.5)	1.44 (1.08, 1.92)	1.22 (0.84, 1.79)	0.83 (0.63, 1.09)	1.41 (0.91, 2.19)
Above college graduate	60.1 (54.1, 66.2) *	66.0 (60.4, 71.6) *	59.5 (53.7, 65.3) *	15.3 (11.2, 19.5) *	2.65 (1.77, 3.95)	1.45 (1.06, 1.97)	1.12 (0.74, 1.71)	2.09 (1.21, 3.61)
Annual Household Income								
<USD 35,000 (ref)	34.8 (29.5, 40.2)	62.3 (56.4, 68.3)	52.0 (46.3, 57.7)	8.1 (5.7, 10.4)	1.0	1.0	1.0	1.0
USD 35,000–USD 49,999	49.5 (39.3, 59.7) *	68.7 (60.0, 77.4)	49.7 (41.5, 58.0)	7.8 (4.5, 11.1)	1.65 (1.04, 2.62)	1.33 (0.82, 2.15)	0.85 (0.56, 1.31)	0.94 (0.52, 1.68)
USD 50,000–USD 74,999	53.5 (46.7, 60.4) *	69.9 (64.4, 75.3)	59.9 (53.7, 66.1)	11.3 (7.3, 15.2)	1.76 (1.24, 2.50)	1.46 (1.05, 2.03)	1.26 (0.88, 1.80)	1.38 (0.77, 2.47)
≥USD 75,000	60.9 (57.2, 64.6) *	66.5 (61.7, 71.4)	60.8 (56.3, 65.3) *	12.2 (9.4, 15.0) *	2.36 (1.76, 3.17)	1.22 (0.88, 1.70)	1.28 (0.94, 1.75)	1.46 (0.93, 2.31)
Did not report	38.2 (34.7, 41.7)	52.8 (48.6, 56.9) *	49.8 (45.5, 54.2)	9.6 (6.3, 13.0)	0.94 (0.57, 1.58)	0.85 (0.53, 1.35)	1.08 (0.65, 1.81)	1.97 (0.95, 4.08)
Insurance status								
Insured (ref)	47.3 (44.3, 50.3)	66.4 (62.9, 69.9)	56.6 (53.7, 59.5)	9.7 (8.2, 11.2)	1.0	1.0	1.0	1.0
Not insured	40.9 (33.3, 48.5)	57.7 (48.1, 67.3)	51.5 (42.8, 60.3)	14.5 (6.3, 22.6)	0.87 (0.64, 1.20)	0.67 (0.44, 1.02)	0.97 (0.69, 1.36)	1.45 (0.79, 2.66)
Prior COVID-19 infection								
Yes	44.0 (40.7, 47.3)	63.8 (59.6, 68.0)	54.8 (51.2, 58.4)	9.4 (6.6, 12.2)	0.70 (0.56, 0.87)	1.04 (0.79, 1.37)	0.96 (0.76, 1.23)	1.0
No (ref)	46.6 (43.3, 49.9)	61.3 (57.9, 64.8)	53.8 (50.3, 57.4)	10.1 (8.1, 12.1)	1.0	1.0	1.0	0.98 (0.65, 1.46)

Note: All values are weighted. Abbreviations: aOR = adjusted odds ratio, CI = confidence intervals, ref = reference. * *p* value < 0.05 in regression model comparing the % of each category (e.g., Non-Hispanic Black) who agree with the reason in the column with the reference category (e.g., Non-Hispanic White) who agree with the reason in that column.

**Table 4:** Distribution of reasons for COVID-19 booster non-vaccination among adults, Household Pulse Survey.

	26 April 2023 to 8 May 2023
	Phase 3.8
	% (95%CI)
Unweighted N	27,223
Perceived vulnerability I think I have enough immunity to COVID-19 from prior doses of the vaccine I am not worried about getting COVID-19 I already had COVID-19	54.9 (53.9, 55.9)
Safety/efficacy concerns I experienced side effects from my previous dose(s) of the COVID-19 vaccine	9.5 (9.0, 10.1)
Lack of cue to action My doctor has not recommended it I am not required to get a COVID-19 booster (by my work or school)	19.0 (18.2, 19.8)

Note: All values are weighted unless otherwise noted. Abbreviations: CI = confidence intervals.

**Table 5:** Changes in reasons for COVID-19 non-vaccination of children, Household Pulse Survey.

	1 September 2021 to 13 September 2021 Phase 3.2 ^a^	29 December 2021 to 10 January 2022 Phase 3.3 ^a^	27 April 2022 to 9 May 2022 Phase 3.4 ^a^	14 September 2022 to 26 September 2022 Phase 3.6	9 December 2022 to 19 December 2022 Phase 3.7	26 April 2023 to 8 May 2023 Phase 3.8	Prevalence Difference ^b^
	% (95%CI)	% (95%CI)	% (95%CI)	% (95%CI)	% (95%CI)	% (95%CI)	% (95%CI)
Unweighted N	2451	5258	4470	5460	8932	7466	
Perceived vulnerability Do not believe children need a COVID-19 vaccine The children in this household are not members of a high-risk group	32.8 (29.2, 36.3)	37.7 (35.5, 39.8)	37.9 (35.4, 40.5)	43.8 (41.4, 46.2)	42.5 (40.1, 44.9)	40.0 (38.0, 42.0)	7.3 (3.4, 11.1)
Safety/efficacy concerns Not sure if a COVID-19 vaccine will work for children Concerned about possible side effects of a COVID-19 vaccine Plan to wait and see if it is safe and may get it later	73.9 (70.9, 76.9)	75.7 (73.4, 77.9)	68.6 (66.4, 70.8)	64.2 (61.7, 66.8)	62.5 (60.5, 64.5)	60.4 (58.2, 62.6)	−13.4 (−16.9, −10.0)
Attitudinal factors/mistrust Do not trust COVID-19 vaccines Do not trust the government	37.8 (34.2, 41.5)	34.3 (31.9, 36.7)	36.5 (33.8, 39.3)	43.6 (41.3, 45.9)	43.7 (41.9, 45.5)	40.3 (38.3, 42.4)	2.5 (−1.2, 6.2)
Logistical barriers ^c^ Concern about missing work to have the children vaccinated Unable to get a COVID-19 vaccine for children in this household Concern about the cost of a COVID-19 vaccine	7.2 (4.3, 10.1)	4.3 (3.4, 5.2)	4.6 (3.3, 5.8)	4.2 (3.4, 5.1)	N/A	N/A	−3.0 (−6.0, 0.0) ^d^
Lack of cue to action The children’s doctor has not recommended it	10.4 (8.2, 12.7)	11.1 (9.5, 12.6)	12.1 (10.3, 14.0)	14.2 (12.6, 15.8)	14.5 (13.0, 15.9)	13.4 (12.4, 14.5)	3.0 (0.5, 5.5)

Note: All values are weighted unless otherwise noted. Abbreviations: CI = confidence intervals. ^a^ Children < 5 not included because they were not eligible for the COVID-19 vaccine. In phase 3.2, children 5–11 were also excluded. ^b^ Difference in prevalence from Phase 3.2 to 3.8. ^c^ This response is only available in the following dataset: 3.2, 3.3, 3.4, 3.6. ^d^ The prevalence difference for logistical barriers was between Phase 3.6 and Phase 3.2.

**Table 6:** Bivariate and multivariable analyses of reasons for COVID-19 non-vaccination of children, Household Pulse Survey, 26 April 2023 to 8 May 2023.

	Bivariate Analysis				Multivariable Analysis			
	Perceived Vulnerability	Safety/Efficacy Concerns	Attitudinal Factors/Mistrust	Lack of Cue to Action	Perceived Vulnerability	Safety/Efficacy Concerns	Attitudinal Factors/Mistrust	Lack of Cue to Action
	% (95%CI)	% (95%CI)	% (95%CI)	% (95%CI)	aOR (95%CI) ^a^	aOR (95%CI) ^a^	aOR (95%CI) ^a^	aOR (95%CI) ^a^
Overall	40.0 (38.0, 42.0)	60.4 (58.2, 62.6)	40.3 (38.3, 42.4)	13.4 (12.4, 14.5)				
Age Groups (in years)								
18–29	46.0 (39.6, 52.3) ^b^	64.0 (57.4, 70.6)	39.1 (33.1, 45.1)	19.0 (14.9, 23.2) ^b^	4.05 (2.47, 6.63)	1.25 (0.75, 2.07)	0.97 (0.55, 1.70)	2.66 (1.23, 5.74)
30–39	42.8 (39.6, 45.9) ^b^	59.9 (56.5, 63.3)	39.5 (36.4, 42.6)	15.2 (13.5, 17.0)	3.17 (2.05, 4.90)	0.86 (0.56, 1.34)	1.57 (0.93, 2.65)	1.95 (1.01, 3.75)
40–49	39.0 (35.9, 42.0) ^b^	59.9 (56.2, 63.6)	40.9 (33.7, 44.1)	10.5 (8.3, 12.6)	2.41 (1.58, 3.65)	0.85 (0.52, 1.37)	1.52 (0.91, 2.54)	1.23 (0.58, 2.57)
50–64	34.2 (29.4, 39.0) ^b^	58.1 (52.6, 63.6)	45.4 (39.7, 51.1)	8.4 (6.3, 10.6)	1.82 (1.12, 2.96)	0.86 (0.51, 1.47)	1.69 (0.91, 3.13)	1.05 (0.51, 2.15)
≥65 (ref)	16.3 (10.9, 21.8)	62.4 (53.3, 71.4)	33.5 (24.4, 42.6)	9.2 (4.4, 14.0)	1.0	1.0	1.0	1.0
Gender								
Men	42.5 (38.8, 46.1) ^b^	56.5 (52.6, 60.5) ^b^	45.4 (41.8, 48.9) ^b^	12.2 (9.9, 14.6)	1.26 (1.06, 1.48)	0.82 (0.67, 1.01)	1.48 (1.20, 1.83)	1.0
Women (ref)	38.2 (36.3, 40.1)	63.9 (61.6, 66.1)	35.6 (33.3, 37.8)	14.3 (12.8, 15.9)	1.0	1.0	1.0	1.08 (0.80, 1.46)
Race/ethnicity								
Non-Hispanic White (ref)	45.5 (43.0, 48.0)	61.1 (58.8, 63.4)	45.4 (43.1, 47.8)	15.0 (13.6, 16.5)	1.0	1.0	1.0	3.09 (1.19, 8.05)
Non-Hispanic Black	26.9 (21.2, 32.7) ^b^	49.9 (42.9, 56.9) ^b^	20.4 (16.7, 24.1) ^b^	10.6 (6.6, 14.6)	0.49 (0.33, 0.74)	0.87 (0.60, 1.27)	0.26 (0.20, 0.34)	3.10 (1.07, 8.97)
Hispanic	30.1 (23.7, 36.5) ^b^	63.1 (56.2, 70.1)	38.1 (30.5, 45.8)	11.6 (8.5, 14.7)	0.51 (0.34, 0.77)	1.16 (0.84, 1.61)	0.58 (0.40, 0.84)	2.27 (0.75, 6.84)
Non-Hispanic Asian	30.3 (19.1, 41.6) ^b^	69.1 (57.8, 80.4)	17.1 (9.3, 24.8) ^b^	6.3 (1.5, 11.1) ^b^	0.44 (0.23, 0.83)	1.32 (0.68, 2.56)	0.31 (0.16, 0.62)	1.0
Non-Hispanic other/multiple races	53.3 (45.7, 61.0)	64.1 (55.5, 72.7)	52.6 (44.5, 60.6)	12.6 (7.0, 18.2)	1.31 (0.86, 1.99)	1.23 (0.80, 1.90)	1.14 (0.84, 1.54)	2.94 (0.84, 10.28)
Highest Educational Attainment								
High school or less (ref)	32.3 (28.1, 36.5)	56.1 (51.9, 60.3)	43.2 (39.6, 46.9)	10.6 (8.6, 12.6)	1.0	1.0	1.0	1.0
Some college or Associate’s	41.5 (38.8, 44.2) ^b^	59.6 (57.0, 62.2)	43.3 (40.7, 45.9)	14.7 (13.0, 16.4) ^b^	1.18 (0.89, 1.57)	1.00 (0.79, 1.26)	1.12 (0.91, 1.37)	1.33 (0.97, 1.82)
College graduate	53.5 (50.7, 56.2) ^b^	69.9 (66.6, 73.1) ^b^	33.7 (30.4, 36.9) ^b^	17.4 (15.1, 19.7) ^b^	1.65 (1.26, 2.14)	1.54 (1.19, 2.00)	0.62 (0.47, 0.81)	1.50 (1.03, 2.16)
Above college graduate	50.1 (46.6, 53.5) ^b^	68.8 (65.0, 72.6) ^b^	26.6 (23.6, 29.7) ^b^	15.7 (13.0, 18.4) ^b^	1.62 (1.15, 2.28)	1.52 (1.08, 2.13)	0.46 (0.35, 0.62)	1.48 (1.05, 2.10)
Annual Household Income								
<USD 35,000 (ref)	26.9 (22.2, 31.7)	63.9 (58.3, 69.4)	42.3 (35.9, 48.8)	10.8 (8.3, 13.2)	1.0	1.0	1.0	1.0
USD 35,000–USD 49,999	36.9 (30.2, 43.6) ^b^	62.2 (54.8, 69.7)	43.1 (35.8, 50.3)	13.5 (8.9, 18.1)	1.35 (0.90, 2.03)	0.88 (0.61, 1.27)	0.89 (0.63, 1.26)	1.22 (0.71, 2.10)
USD 50,000–USD 74,999	41.1 (35.2, 46.9) ^b^	67.4 (62.3, 75.5)	44.8 (38.1, 51.5)	14.4 (11.0, 17.8)	1.48 (1.00, 2.19)	1.09 (0.75, 1.60)	0.93 (0.60, 1.44)	1.30 (0.90, 1.88)
≥USD 75,000	51.6 (48.3, 54.9) ^b^	65.1 (62.0, 68.1)	39.7 (36.5, 43.0)	16.0 (13.8, 18.1) ^b^	1.94 (1.43, 2.64)	0.89 (0.65, 1.21)	0.77 (0.56, 1.05)	1.32 (0.94, 1.86)
Did not report	34.1 (30.7, 37.4) ^b^	49.7 (45.8, 53.6) ^b^	37.1 (33.4, 40.7)	11.4 (9.2, 13.6)	1.45 (0.95, 2.20)	0.82 (0.53, 1.26)	0.98 (0.64, 1.49)	1.20 (0.68, 2.12)
Insurance status								
Insured (ref)	43.1 (41.0, 45.3)	66.1 (63.8, 68.5)	41.0 (38.7, 43.4)	14.6 (13.3, 15.9)	1.0	1.0	1.0	1.73 (1.12, 2.68)
Not insured	32.6 (22.4, 42.8)	49.3 (39.7, 58.9) ^b^	50.9 (41.1, 60.7)	9.6 (5.1, 14.1)	0.76 (0.49, 1.20)	0.57 (0.39, 0.82)	1.30 (0.88, 1.92)	1.0
Prior COVID-19 infection								
Yes (ref)	43.8 (41.2, 46.4)	66.4 (63.7, 69.0) ^b^	41.8 (39.1, 44.6)	14.6 (13.2, 15.9)	1.03 (0.86, 1.22)	1.38 (1.13, 1.67)	1.03 (0.86, 1.22)	0.95 (0.76, 1.20)
No	37.1 (34.3, 39.8) ^b^	55.4 (52.0, 58.8)	41.1 (37.7, 44.4)	12.6 (10.6, 14.6)	1.0	1.0	1.0	1.0

Note: All values are weighted. Abbreviations: aOR = adjusted odds ratio, CI = confidence intervals, ref = reference. ^a^ Adjusted for age, gender, race/ethnicity, highest educational attainment, annual household income, insurance status, and prior COVID-19 infection. ^b^ *p* value < 0.05 in a regression model comparing the % of each category (e.g., Non-Hispanic Black) who agree with the reason in the column with the reference category (e.g., Non-Hispanic White) who agree with the reason in that column.

## Data Availability

The data that support the findings of this study are openly available at https://www.census.gov/programs-surveys/household-pulse-survey/datasets.html.
